# Supplementary material for: Permo–Triassic boundary carbon and mercury cycling linked to terrestrial ecosystem collapse
Source: Nat Commun. 2020 Jun 11;11:2962. doi: 10.1038/s41467-020-16725-4 (PMC7289894; doi:10.1038/s41467-020-16725-4)
Supplement: Supplementary file 1 — Supplementary Information [file 41467_2020_16725_MOESM1_ESM.pdf]

**Supplementary Information for "Permo-Triassic boundary carbon and mercury cycling linked to terrestrial ecosystem collapse" by Jacopo Dal Corso et al.**

## Supplementary Note 1

We explore a third scenario in Supplementary Figure 1 where instead of adding a biosphere pulse to scenario I, we instead add a pulse of additional volcanism. The timeframe is assumed to be the same as the biosphere pulse and the magnitude is chosen to match the Hg record in panel C. This scenario can produce a Hg/TOC record that agree with data, however the $\delta^{202}$Hg record does not show any decline in shallow water as the Hg source remains atmospheric, and the required increase in Hg degassing is extreme and around an order of magnitude more than required in scenario II. This is because an atmospheric Hg signal is much more diluted when it reaches nearshore sediments than a riverine signal. Similarly, this scenario fails to reproduce the nadir in carbonate $\delta^{13}$C.

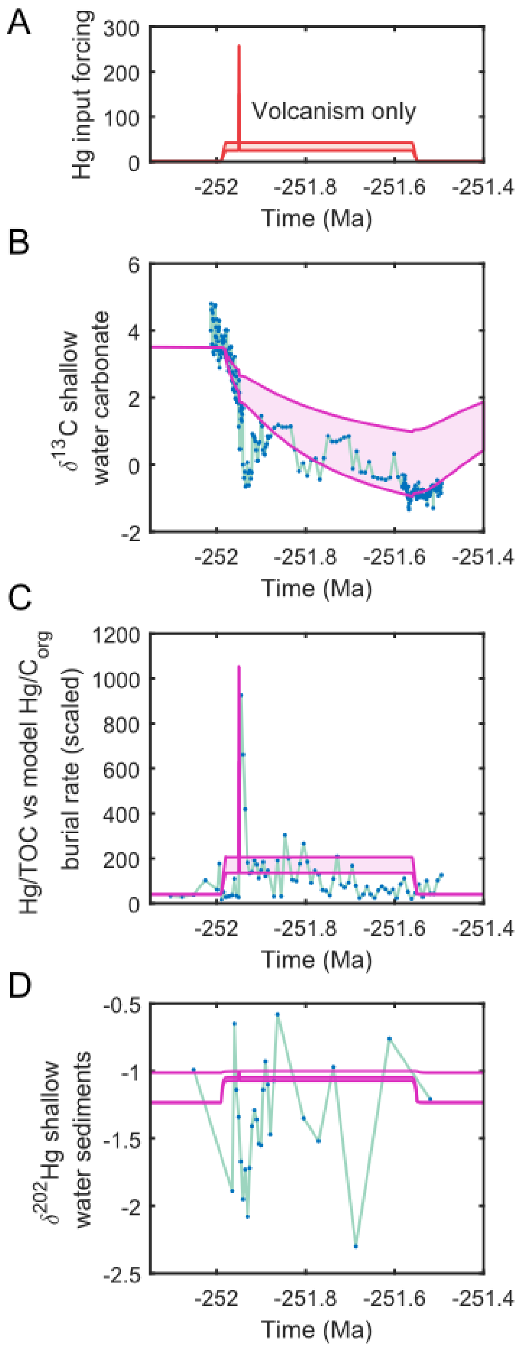


**Supplementary Figure 1. Model results under Scenario I – 2.** Top panel (A) shows input forcing in terms of relative rate of Hg input, next panel (B) shows $\delta^{13}C$ of new shallow ocean carbonate, third panel (C) shows Hg/TOC data versus the model molar Hg/$C_{org}$ burial rate, which has been scaled to match the background as the model does not calculate weights. Final panel (D) shows model $\delta^{202}Hg$ against data, where solid line is shallow water (box s) and dashed line is deep water (box d).
